# Step-by-Step Design of New Theranostic Nanoformulations: Multifunctional Nanovectors for Radio-Chemo-Hyperthermic Therapy under Physical Targeting

**DOI:** 10.3390/molecules26154591

**Published:** 2021-07-29

**Authors:** Shoeb Anwar Ansari, Eleonora Ficiarà, Federico D’Agata, Roberta Cavalli, Lucia Nasi, Francesca Casoli, Franca Albertini, Caterina Guiot

**Affiliations:** 1 Department of Neurosciences, University of Turin, 10124 Turin, Italy; shoebanwarmohammedkhawja.ansari@unito.it (S.A.A.); federico.dagata@unito.it (F.D.); caterina.guiot@unito.it (C.G.); 2 Department of Drug Science and Technology, University of Turin, 10125 Turin, Italy; roberta.cavalli@unito.it; 3 IMEM CNR, Parco Area delle Scienze 37/A, 43124 Parma, Italy; lucia.nasi@imem.cnr.it (L.N.); francesca.casoli@imem.cnr.it (F.C.); franca.albertini@imem.cnr.it (F.A.)

**Keywords:** nanostructure, oxygen, SPIONs, magnetic driving, theranostic

## Abstract

While investigating the possible synergistic effect of the conventional anticancer therapies, which, taken individually, are often ineffective against critical tumors, such as central nervous system (CNS) ones, the design of a theranostic nanovector able to carry and deliver chemotherapy drugs and magnetic hyperthermic agents to the target radiosensitizers (oxygen) was pursued. Alongside the original formulation of polymeric biodegradable oxygen-loaded nanostructures, their properties were fine-tuned to optimize their ability to conjugate therapeutic doses of drugs (doxorubicin) or antitumoral natural substances (curcumin). Oxygen-loaded nanostructures (diameter = 251 ± 13 nm, ζ potential = −29 ± 5 mV) were finally decorated with superparamagnetic iron oxide nanoparticles (SPIONs, diameter = 18 ± 3 nm, ζ potential = 14 ± 4 mV), producing stable, effective and non-agglomerating magnetic nanovectors (diameter = 279 ± 17 nm, ζ potential = −18 ± 7 mV), which could potentially target the tumoral tissues under magnetic driving and are monitorable either by US or MRI imaging.

## 1. Introduction

In spite of the dramatic progress in tumor control, some specific organs and/or tumor stages require multi-treatment approaches to counteract cancer progression. This is the case, for instance, in unresectable head and neck tumors, which are normally treated with chemoradiotherapy and also benefit from external hyperthermic treatments [1,2]. Moreover, pancreatic cancer normally requires multimodal treatments since the response to traditional therapies is largely unsuccessful [3].

Most importantly, tumors of the central nervous system (CNS) are still poorly treated because, very often, it is almost impossible to reach their site without seriously damaging the nearby structures. 

Besides surgery, less invasive approaches are also critical, mainly due to the presence of biological membranes (such as the blood–brain barrier, BBB) preventing the passage of drugs [4,5,6].

As far as conventional radiotherapy is concerned, most of the tumor tissues are weakly radiosensitive, also because of their hypoxic state.

The main therapeutic approaches are scarcely effective when applied individually; therefore, it may be useful to investigate whether their combination may induce synergistic effects. 

Consequently, we developed a multimodal approach to target all the above critical issues with a unique theranostic system.

### 1.1. Loading and Delivery of Oxygen to Hypoxic Tissues

The feasibility of oxygen delivery to hypoxic tissues and organs has been investigated in various studies [7,8]. Hemoglobin-loaded particles (Hb particles), encapsulated within a biodegradable polymer, have been prepared for use as oxygen carriers [9] and showed a oxygenation capacity similar to that of native hemoglobin, as well as a prolonged circulation time after administration, in mice. Albumin microbubbles for oxygen and nitrogen delivery have been formulated [10] for use as contrast agents, together with lipid-coated perfluorocarbon (PFC) microbubbles, which were tested in a model of anemic rats [11,12], allowing survival at very low hematocrit levels. 

Due to their excellent solubility for oxygen, perfluorocarbons were used also in the so-called ‘liquid-assisted ventilation’ technique, where PFC recruitment of surfactant-deficient (e.g., neonatal hyaline membrane disease) or impaired alveoli was shown to be very effective [13]. 

Reducing the dimension of the bubble allows larger internal partial pressures due to Laplace’s law, and oxygen, being very soluble in perfluorocarbons both storing and delivering oxygen in hypoxic tissues, is enhanced by nanodimensions: oxygen-loaded nanobubbles have therefore been developed. Because of their theranostic property, oxygen-loaded nanobubbles are also very appealing nanocarriers for potential application in cancer therapy.

### 1.2. Loading and Delivery of Anticancer Drugs 

Interestingly, the choice of vectors in the nanometric scale is challenged by the presence of very selective biological barriers [14] or when the specific position is difficult to target. Due to their small size, nanoparticles have distinct properties compared with the bulk form of the same materials, and the interactions of nanoscale objects with living systems have yet to be thoroughly investigated. For instance, in biological fluids, proteins interact with nanoparticles, inducing in vivo responses [15], which may limit the efficiency of the systemic delivery of drugs and active compounds expected to reach a specific organ. As a matter of fact, nanocarriers can improve the drug’s transport efficiency and targeting of the cancer cells and, acting at a specific site, they can reduce the side effects on the body. Significant improvements in the effectiveness of treatment by ‘nano-carried’ or ‘bulk’ drugs are, however, debatable. As gene therapy is a new frontier in cancer care, the use of (non-viral) nanovectors for genetic material delivery is very promising [16].

To improve the local effectiveness, many targeting systems have been devised, mainly based on the actual bias concerning the coupling of targeting substances on the surface of the nanostructure.

In addition to chemical ones, physical targeting methods have also been investigated in the past [17]. Ultrasounds (US), in an appropriate frequency and intensity range, were shown to be able to transiently open the BBB, allowing the safe and effective delivery of drugs to the CNS. Based on this concept, a technique called Focused Ultrasound (FUS) has been developed, and many applications, mainly performed under MRI control, have already been demonstrated under FDA permission [18].

Details on the US-mediated oxygen release can be found in the literature [19]. Such physical enhancement is of great importance whenever tissues can be effectively sonicated, e.g., the depth of the lesion to be treated is not too large and there are no interposed tissues with high impedance. For this reason, sonication is scarcely of interest for application in the CNS since the skull almost completely shields the mechanical waves.

### 1.3. Magnetic Physical Targeting and Hyperthermia

Another physical targeting method near to maturity is based on the use of magnetic fields able to drive nanoparticles with proper magnetic properties to their site of action. Many papers have focused on the feasibility of external driving implants [20], on the biodistribution of the magnetic vectors [21], on their ability to overcome the BBB [22] and on their toxicological properties [23,24].

SPION-decorated dextran-sulfate-shelled OLNBs, i.e., magnetic oxygen-loaded nanobubbles (MOLNBs), manufactured by adding SPIONs to the surfaces of polymeric nanobubbles, have been designed as promising theranostic carriers for delivering oxygen and chemotherapy to critical tumors, such as CNS ones. Physicochemical and cytotoxicological properties and in vitro internalization by human brain microvascular endothelial cells, as well as the motion of MOLNBs in a static magnetic field, have therefore been investigated [25]. Previous studies showed the potentiality of MOLNBs to be magnetically drivable using external permanent magnets [25]. A challenging goal could be tailoring the driving magnetic field based on the position and dimension of the tumor and the membranes to be crossed.

Hyperthermia, a treatment designed to raise the temperature of cancerous regions of the body to 40–43 °C, can cause cancer cell death by enhancing the cytotoxic effects of radiotherapy and chemotherapy. Although it is rarely used as a single treatment method in contemporary oncological management, several randomized studies show that, in conjunction with radiotherapy, it has the potential to improve the results of concomitant cancer treatments without significantly increasing their toxicity [26].

Magnetic hyperthermia, consisting of the endogenous production of heat from nanomagnetic structures precisely inserted into the target tumor by applying an external time-varying magnetic field, is one of the most efficient and safe therapeutic approaches [27].

Details on the Materials and Methods are given in the Section 4.

## 2. Results

### 2.1. Oxygen-Loaded Nanosystems: Loading and Delivery of Oxygen to Hypoxic Tissues

**Figure 1:**
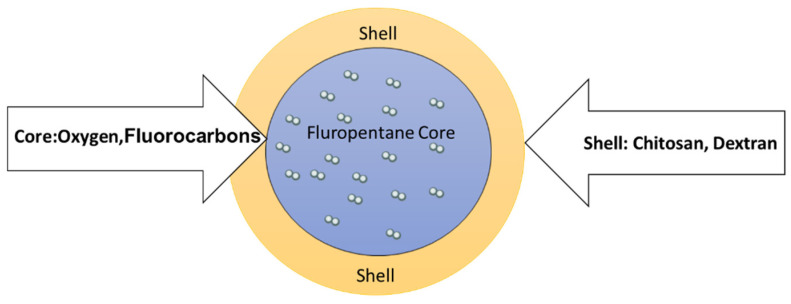
Structure of the oxygen-loaded nanosystem.

Different oxygen-loaded nanosystems have been studied by our team, differing both for the polymer in the shell and the inner fluorocarbon agent storing oxygen to enhance the structure stability. A detailed description is given in Table 1 and an illustration in Figure 1.

The main difference between PFP and DFP is their boiling point (28 °C for the former, in vapor state at human body temperature, therefore forming bubbles, and 55 °C for the latter, which is liquid at body temperature, forming droplets). This difference in boiling points might also impact the echogenic properties and the possibility of detection by US. Both PFP and DFP are suitable for human use and are expected to be completely eliminated by expiration. Furthermore, all the natural polysaccharide polymers used in the different formulations are biodegradable and biocompatible. 

According to the manufacturing details given in Section 4, oxygen-loaded nanosystems can be characterized by the natural polysaccharide used for the shell, which defines their surface properties, their ζ potential and the oxygen exchange effectiveness. For the cases of chitosan and dextran, details can be found in [28,29]. 

**Figure 2:**
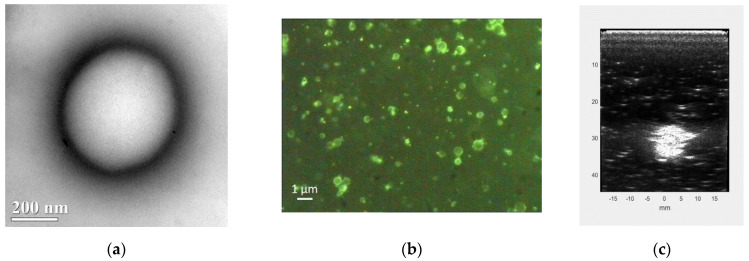
TEM (**a**), optical fluorescence (**b**) and US (**c**) imaging of OLNBs.

Due to their dimensions and physical characteristics, the morphology of the above nanostructures can be investigated by transmission electron microscopy (TEM) (Figure 2a), fluorescent microscopy provided an FITC-labeled shell has been applied (Figure 2b) and, especially for OLNBs, by US (Figure 2c).

Precise quantification of the oxygen contained in the nanostructures is very difficult; however, indirect estimates, e.g., based on salt oxidation, give an average value of 400 μg/mL for OLNBs [30]. Besides their composition, oxygen release kinetics from the OLNBs by passive diffusion depend also on the characteristics of the considered external medium, its temperature and its initial oxygen concentration. Moreover, the rate and extent of the oxygen release can be significantly increased by sonication [29,30] (see Supplementary Materials, Figure S1). 

**Figure 3:**
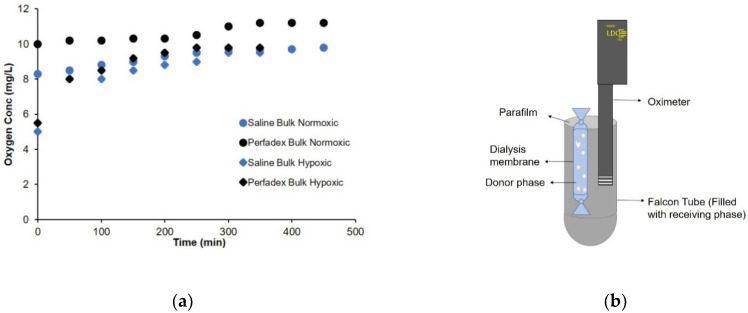
Examples of passive oxygen release in fluids: (**a**) oxygen concentration in different liquids, saline and Perfadex, in normoxic and hypoxic bulk at 4 °C; (**b**) illustration of the device for measuring oxygen diffusion from the donor compartment to the bathing medium. Data were measured with accuracy of ±1.5% of the nominal value.

In Figure 3, the rate of oxygen exchange between the donor compartment containing the OLNBs and the receiving compartment composed of different liquids, i.e., saline and Perfadex^®^, used as perfusion medium in lung transplants, is shown (Figure 3a), together with the experimental device designed for the measurement (Figure 3b) (details given in Section 4).

According to the results presented in Figure 3, oxygen exchange occurs by passive diffusion and is therefore accelerated in hypoxic media. The coefficient of diffusion is markedly dependent on the characteristics of the external media. 

Preliminary experiments of oxygen diffusion were performed on animal models using dextran-shelled OLNDs (see Supplementary Materials). 

### 2.2. Drug-Loaded Oxygen-Loaded Nanosystems: Loading and Delivery of Anticancer Drugs

**Figure 4:**
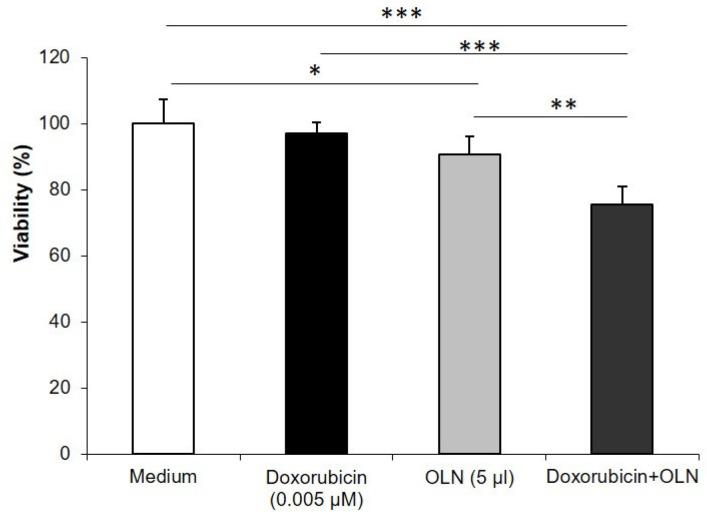
DOX in OLND promotes synergistic antitumor effects. Cell viability was evaluated by MTT assay. Data are expressed as percentage vs. control. TUBO cells were left untreated or incubated for 48 h with DOX (0.005 μM), OLNDs (2.5% *v*/*v*) or DOX-loaded OLNDs (0.005 μM/2.5% *v*/*v*). Results are shown as means ± SD from three independent experiments. Significance of the differences: * *p* < 0.05; ** *p* < 0.005; *** *p* < 0.001.

Oxygen-loaded nanodroplets (OLNDs) can be loaded with drugs and/or natural substances to counteract the development of tumors or prevent their local recurrence. Doxorubicin (DOX) and curcumin (Curc) have been previously investigated in this context [31,32]. Doxorubicin is easily encapsulated in the chitosan-shelled nanodroplet structure and in DOX-loaded OLNDs, showing, even at low doses, marked effectiveness when administrated both in vitro and in vivo to TUBO cells, in a cloned rat Her2/neu+ cell line established from lobular carcinoma of a BALB-neuT mouse (Figure 4). Internalization of DOX-loaded OLNDs occurred also in vivo after administration in tumor-bearing mice, and the tumor growth limitation is related to the variable uptake efficiency of the nanovector [31]. 

The in vivo results confirmed the potential of OLNDs as oxygen and drug delivery systems [31] after administration in mice.

**Figure 5:**
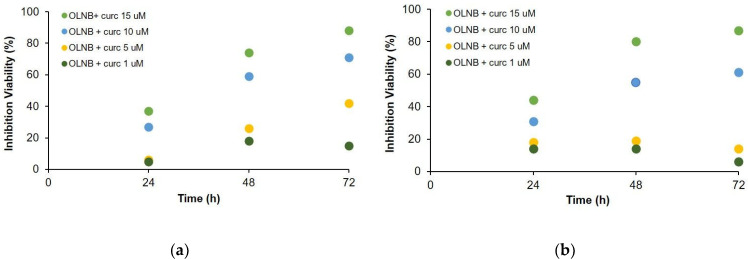
Percentage of inhibition of viability: (**a**) PC-3 tumor cells; (**b**) DU-145 tumor cells.

Another formulation based on the association of dextran sulfate-shelled OLNBs and curcuminoids (Curc) was shown to be effective in vitro against prostate cancer cells. The inhibition of the viability of PC-3 and DU-154 tumor cells is greatly improved when Curc is delivered by OLNBs at all doses (see Figure 5). Moreover, other important inhibitory effects of Curc-loaded OLNBs on cell motility and adhesivity were detected [32].

### 2.3. SPION-Decorated OLNBs: Manufacturing and Physicochemical Characterization of MOLNBs

**Figure 6:**
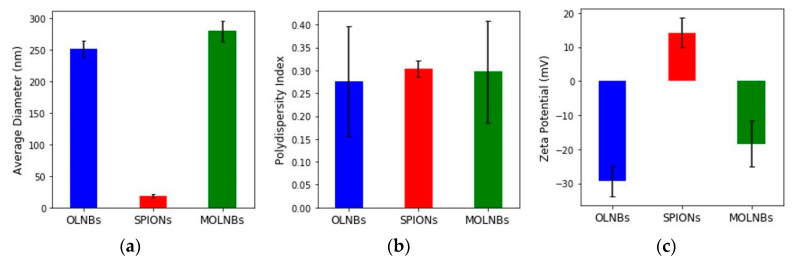
Physicochemical characteristics of nanocarrier formulations: (**a**) average diameter (nm); (**b**) polydispersity index; (**c**) ζ potential (mV) for OLNBs (blue), SPIONs (red) and MOLNBs (green).

**Figure 7:**
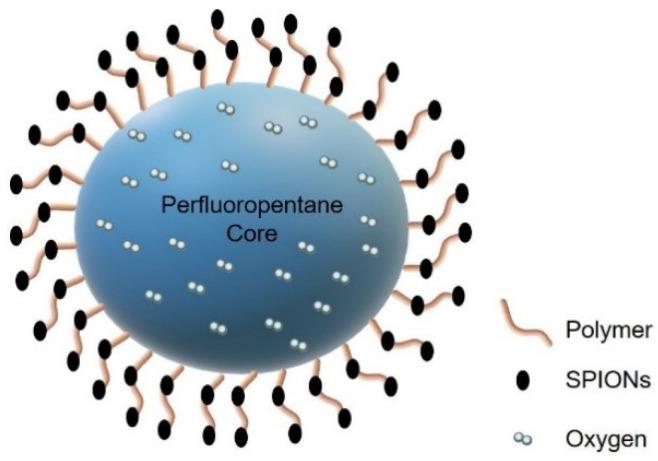
Illustration of MOLNBs (negatively charged dextran shell NBs decorated with positively charged SPIONs by electrostatic interaction).

The physicochemical characterization of OLNBs and MOLNBs is reported in Figure 6. A scheme of MOLNB is given in Figure 7. The highly negative ζ potential (around -29 mV) of OLNBs is due to the presence of dextran sulfate on the nanobubble surface. The average ζ potential of MOLNBs decreased to -18.33 mV because of the electrostatic interaction between the positively charged SPIONs decorating the negatively charged OLNBs. The pH values of OLNBs, SPIONs and MOLNBs were 6.6, 7.3 and 6.8, respectively.

Other crucial parameters of the MOLNB nanosuspension, such as viscosity and osmolarity, were 0.96 cP and 354 mOsm, respectively. These values, together with their non-hemolytic activity [25], indicate that they are suitable for administration in animal models.

**Figure 8:**
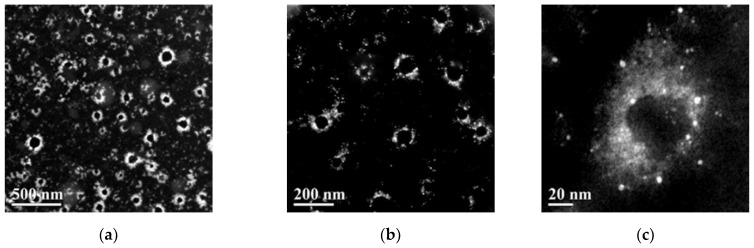
HAADF-STEM images of the MOLNBs decorated with SPIONs taken at different magnifications ((**a**) = 500 nm, (**b**) = 200 nm, (**c**) = 20 nm).

The MOLNB structure can be visualized in high-angle annular dark-field imaging (HAADF-STEM) mode at different scales (Figure 8). The atomic number contrast images clearly show the core/shell structure of the NBs and the SPIONs (bright spots) embedded in the polymeric shell of MOLNBs.

**Figure 9:**
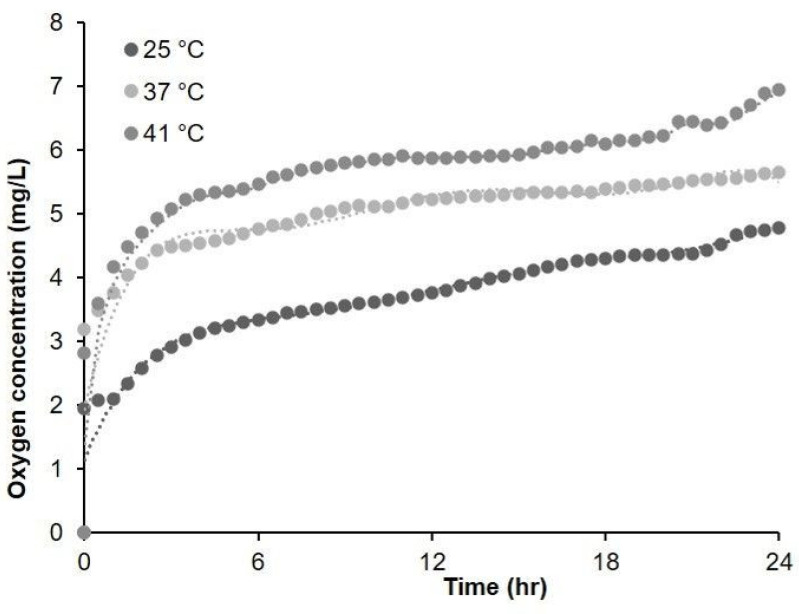
Oxygen release study performed by passive diffusion in saline solution at different temperatures over 24 h. Data of oxygen concentration were measured with accuracy of ±1.5% of the nominal value.

Oxygen release at different temperatures (Figure 9) was evaluated, showing a significant increase at hyperthermic temperatures. In fact, when stored at 4 °C, MOLNBs were stable for up to 90 days, and their ζ potential slightly changed from (−18.3 ± 2.8 mV) to (−16.2 ± 1.7 mV), confirming the chemico-physical stability of the nanostructure.

### 2.4. Magnetic Characterization of MOLNBs 

#### In Vitro MRI Test

The properties of MOLNBs as contrast agents were investigated in vitro by MRI scanning using clinical equipment (3.0 T, Vida, Siemens), evaluating their relaxation time at different concentrations of the decorating SPIONs by transverse relaxation (T_2_) mapping. T_2_ values were obtained from exponential fitting of the signal and TE and are shown in Table 2, which indicates that an increasing concentration of SPIONs led to a T_2_ reduction (see Supplementary Materials, Figure S2). 

### 2.5. Magnetic and Hyperthermic Properties of MOLNBs 

The magnetic properties of OLNBs, SPIONs and MOLNBs were measured at room temperature by an alternating gradient force magnetometer (AGFM) by applying a maximum magnetic field μ_0_H = 1.8 T. 

**Figure 10:**
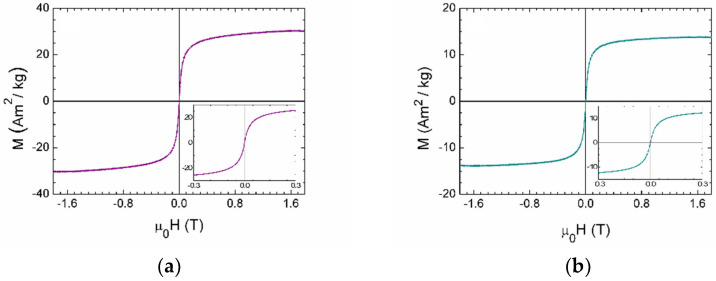
(**a**) Magnetization cycle of SPIONs; (**b**) magnetization cycle of MOLNBs. In the insets, a zoom of the measurements is reported, highlighting the superparamagnetic behavior of the samples (i.e., absence of magnetic hysteresis and remanence).

The sample of OLNBs showed a weak diamagnetic signal, obviously negligible with respect to that of SPIONs and MOLNBs, whose magnetic cycle is, respectively, described in Figure 10a,b. 

In Figure 10a, the magnetization cycle of SPIONs is reported. It showed a superparamagnetic behavior, with a magnetization value of 30 Am^2^/kg at the maximum applied field μ_0_H = 1.8 T.

Furthermore, the MOLNB sample showed superparamagnetic behavior, with a specific magnetization value of 14 Am^2^/kg at the maximum applied field μ_0_H = 1.8 T, which is compatible with the known mass concentration of SPIONs (i.e., around 40%) in MOLNBs when taking into account the experimental error of the magnetic measurements. In conclusion, these results confirm that MOLNBs show superparamagnetic behavior with the magnetic moment expected for a specific quantity of incorporated SPIONs.

According to these magnetic properties, MOLNBs are effective candidates for hyperthermic agents [33].

**Figure 11:**
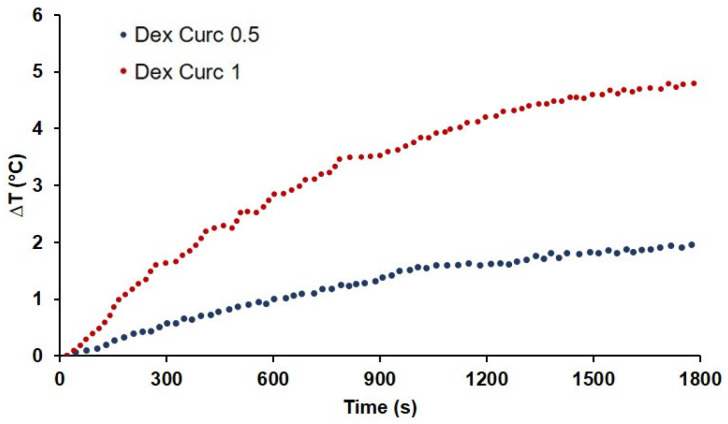
Magnetic hyperthermia measurements under an alternating (f = 429 kHz) magnetic field of 300 Oe for 30 min: temperature increase as a function of time of the curcumin-loaded dextran MOLNBs at different concentrations of SPIONs (1 mg/mL and 0.5 mg/mL).

The hyperthermic experiment was carried out by exposing a colloidal aqueous suspension of curcumin-containing MOLNBs to an RF magnetic field for 30 min. In Figure 11, the time dependence of the temperature of the samples under irradiation is reported for MOLNBs with two different (low) concentrations of SPIONs (0.5 and 1 mg/mL). Moreover, NBs without SPIONS were exposed following the same procedure in order to evaluate the effects of the thermal dispersion of the equipment (which was subjected to the MOLNB temperatures). After 30 min of exposure, the temperature increase in the curcumin-loaded MOLNBs containing 0.5 mg/mL and 1 mg/mL SPIONs ranged between 2 and 4.8 °C and the corresponding SAR value ranged between (9 ± 1) and (19 ± 1) W/g, respectively.

The increase in the temperature of MOLNBs is obviously tunable by properly tailoring the nanobubbles and SPION concentration, the RF power and its timing in order to reach the hyperthermic temperature required by the clinical application.

The curcumin concentration in the solution, after filtering the curcumin-loaded MOLNBs, was evaluated by HPLC and no significant difference was detected before and after HT.

### 2.6. Drivability by the Application of Weak Static Magnetic Fields of MOLNBs

We proposed a setup aimed to simulate the motion of MOLNBs inside fluids in the presence of magnetic fields in different configurations (which can be positioned, for instance, on the skull for CNS tumors), monitoring by US (see Supplementary Materials). 

**Figure 12:**
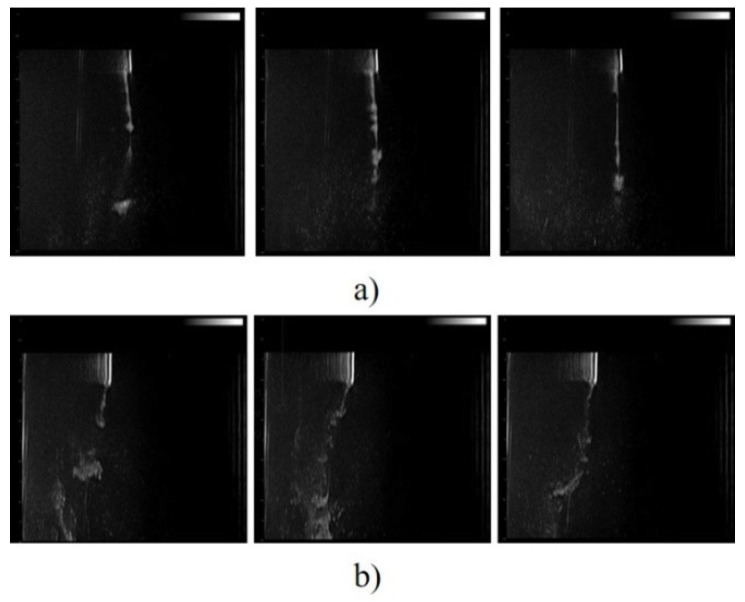
US imaging snapshot of MOLNBs in the absence (**a**) and the presence (**b**) of the magnetic field generated by two magnets (distance 4 cm from each other). Images were recorded at different time frames (5, 15, 30 s) from the injection.

After injecting MOLNBs in the setup under US and monitoring the effect of the magnetic field, a significant deviation in MOLNBs’ motion toward the magnets was observed (Figure 12b) with respect to the homogeneous flow of MOLNBs in the vertical direction in the absence of a magnetic field (Figure 12a).

## 3. Discussion

Our team developed a multifunctional theranostic nanovector able to act as an oxygen reservoir for delivering to the hypoxic site of interest (e.g., the tumor) the amount of oxygen required to optimize the effect of radiotherapy, in controlled and prolonged kinetics. Due to the presence of gases or vaporizable compounds within the inner core (oxygen and fluorocarbons), these nanovectors are called oxygen-loaded nanobubbles (OLNBs) or oxygen-loaded nanodroplets (OLNDs) depending on the vapor or liquid status of the core.

While their shells, made of phospholipids, polymers and surfactants, stabilized the structures, because of their gas cores, they were echogenic and could be used as contrast agents in ultrasonic and photoacoustic imaging. These bubbles, which have novel properties and flexible structures, can be engineered in a variety of sizes as vehicles for gas and drug delivery applications [34]. 

The main characteristics of oxygen-loaded nanostructures manufactured with different natural biocompatible and biodegradable polymers containing different perfluorocarbons are revised looking at their possible application as radiosensitizers, based on previous in vivo experiments of oxygen diffusion monitored by transcutaneous oximetry (tcpO2) and by photoacoustic imaging on mouse legs, which demonstrated a sustained oxygenation effect of OLNDs for up to 1 h [30].

Their ability to carry and deliver anticancer agents is discussed as well. The cell viability studies confirmed that these multifunctional nanovectors can act as drug delivery systems, improving the effectiveness of the encapsulated drugs, e.g., DOX in OLNBs, which promoted a synergistic antitumor effect, and curcumin-loaded OLNBs, which improved the inhibition of tumor cell viability. Although our evidence is based on two cases, the nanosystems are very flexible and could be easily designed for administrating a large variety of different drugs.

Adding to the above nanostructures specific magnetic properties greatly enhances their potentiality either for diagnosis (MRI imaging, in addition to US), therapy (hyperthermia) or the possibility of physical targeting of tissues by exploiting the magnetic driving.

Other indirect advantages are also expected, e.g., the temperature in the hyperthermic range induces a marked change in oxygen release over time, with increases in the external aqueous solution oxygen concentration of up to 0.5–1 mg/L. This oxygenation effect might further enhance the therapeutic outcome when hyperthermia is combined with radiation therapy.

By gradient force magnetometry, we confirmed the superparamagnetic behavior of our nanosystems, with the magnetic and hyperthermic properties being correlated with the small size of the MOLNBs and the average number of decorating SPIONs.

In particular, we detected a specific magnetization of 30 Am^2^/kg for SPIONs, and following the decoration of the OLNBs (without any intrinsic magnetic property) with SPIONs at 40% in mass concentration, the final value of 14 Am^2^/kg for MOLNBs is acceptable within the experimental errors. The specific magnetization can be finely tuned by varying the mass concentration of SPIONs according to any specific application, i.e., the position and characteristics of the target and of the driving magnetic field.

The SPION concentration also defines the hyperthermic efficiency of MOLNBs, as well as the increase in temperature required by the therapy to induce cellular damage or to promote tumor oxygenation in combination with radiotherapy (or possibly also to enhance the penetration across biological membranes such as the BBB).

Curcumin-loaded MOLNBs with different concentrations of SPIONs (0.5 and 1 mg/mL) showed a mild heating response to HT treatment, increasing by 2 °C and 4.8 °C, respectively, after 30 min.

Moreover, the curcumin concentration before and after HT was not significantly different, indicating that no disruption or shrinking occurred during their heating. This is an important result demonstrating safe and long-lasting drug delivery also under HT treatment.

Figure 12 showed that by applying an external magnetic field generated by permanent magnets, it is possible to induce a significant deviation in the motion of the MOLNBs towards the magnets. This behavior might be exploited for targeted drug delivery.

Interestingly, the possibility of US monitoring may be useful for triggering drug release from MOLNBs. In combination with MRI, it may allow MOLNBs to be used for precision medicine applications.

As a matter of fact, although such in vitro investigations are very promising, the above hypotheses need accurate validation on in vivo animal models in order to understand the capability of MOLNBs to reach the critical tumors that they intend to target. For instance, their main weakness for application to CNS tumors is that we have presently no data concerning the in vivo BBB trespassing of MOLNBs. Although our preliminary measurements seem to exclude relevant cell toxicity and hemolytic activity [25], the possibility of systemic administration and BBB overcoming should be tested, to better understand the final fate of MOLNBs.

In fact, BBB crossing is another very challenging task: although such an ability of nanovectors similar to MOLNBs has been shown in the literature [14], specific interactions between CNS cells and MOLNBs need to be more deeply understood. Interestingly, the behavior and safety of magnetic nanoparticles and their motion in brain tissue under a magnetic field were previously investigated, showing no adverse effects associated with their motion [35]. In addition, innovative approaches to characterize the BBB permeability and the interaction with nanoparticles can be found in the literature [36], highlighting the crucial importance of computational models to tune an optimal magnetic force-field.

All the collected results show that MOLNBs are a versatile theranostic tool whose strengths are their multi-imaging capability and their ability to physically trigger the delivery of active molecules in combination with therapy protocols. 

Thus, MOLNBs could represent a promising nanoplatform for applications to fulfil non-trivial therapeutic needs, such as those related to head and neck tumors, pancreatic tumors and CNS pathologies. 

Finally, our results seem to encourage the possibility of non-systemic administration approaches, e.g., the feasibility of some magnetic driving of the MOLNBs from the brain ventricles (filled by the cerebrospinal fluid (CSF), in which MOLNBs could be injected from the intravertebral spaces) to the nearby tumors to which the cargoes have to be delivered. The contact of CSF with the CNS makes it an attractive medium for drug delivery, circumventing systemic barriers. Several studies demonstrated the possibility of the intrathecal administration of nanoparticles [37] and the administration of active agents directly into the ventricles [38].

Such a driving approach would require a ‘personalized’ model, based on CT or MRI imaging and a grid of permanent magnets producing the required magnetic field. However, some of the difficulties can be overcome by the fact that MOLNBs, being a theranostic system, are monitorable either via MRI or US sonography, allowing a real-time tracing of their path.

In conclusion, although still speculative, the availability of versatile theranostic nanovectors such as MOLNBs could open new scenarios for future integrated therapies.

## 4. Materials and Methods

Ethanol, perfluoropentan, Fe^2+^ and Fe^3+^ were from Sigma-Aldrich. Epikuron^®^ 200 (soy phosphatidylcholine 95%) was a kind gift from Cargill. Palmitic acid and dextran sulfate sodium salt (Mw = 100,000) were from Fluka (Buchs, CH, Switzerland). 

### 4.1. Preparation of Chitosan Oxygen-Carrying Nanobubbles

At 25 °C, a saturated solution of O_2_ in 0.9 mg/mL NaCl was prepared and used for nanobubble preparation. Then, 300 µL of an ethanolic solution containing Epikuron 200 (1%, *w*/*v*) and palmitic acid (0.3%, *w*/*v*) was injected into a sealed vial of 10 mL of the O_2_ solution under stirring. The system was homogenized for 2 min at 20,000 rpm using an Ultra-Turrax TA 18 homogenizer (IKA), which allowed the tool head to work at high peripheral speeds to generate very high shear rates for fast and efficient sample processing. Then, under continuous oxygen purging, 400 µL of 2.5% chitosan solution in acetate buffer with pH 5 was added as a polymeric shell for the nanobubbles. Then, 1 N NaOH solution was added under gentle stirring to obtain the desired pH.

### 4.2. Dextran and Dextran Sulfate Oxygen-Loaded Nanodroplets

For oxygen-loaded nanodroplet liquid formulations, 1.5 mL DFP (Fluka, Buchs, Switzerland) along with 0.5 mL polyvinylpyrrolidone (Fluka, Buchs, Switzerland) and 1.8 mL soy lecithin (Degussa, Hamburg, Germany) solved in 1% *w*/*v* ethanol (Carlo Erba, Milan, Italy) and 0.3% *w*/*v* palmitic acid solution (Fluka, Buchs, Switzerland) were homogenized in 30 mL water (preparation A) or phosphate-buffered saline (PBS) (preparations C-D) for 2 min at 24,000 rpm by using an Ultra-Turrax SG215 homogenizer (IKA, Staufen, Germany). Ultrapure water was obtained using a 1–800 Millipore system (Molsheim, France). Thereafter, the solution was saturated with O_2_ for 2 min. Finally, 1.5 mL dextran sulfate solution was added dropwise and homogenized for 2 min at 13,000 rpm. PFP was used as a core fluorocarbon in the OLNB water formulation. Without adding oxygen, oxygen-free nanodroplet (OFND) and nanobubble (OFNB) water formulations were produced according to OLND and OLNB methods. The OLND preparation process was used for the oxygen-saturated solution (OSS) water formulation, skipping the inclusion of dextran and DFP. Core fluorocarbon without adding oxygen, oxygen-free nanodroplet (OFND) and nanobubble (OFNB) water formulations were produced according to OLND and OLNB methods. 

### 4.3. Synthesis of SPIONs

There are numerous methods for synthesizing SPIONs. The co-precipitation method is the simplest chemical method for producing iron oxide nanoparticles. Using this method, we dissolved 0.99 g and 2.7 g of Fe^2+^ and Fe^3+^ in 50 mL of distilled water, respectively. Magnetite is prone to oxidation; therefore, inert gas (nitrogen) purging was required under continuous stirring at 85 °C for 45 min to prevent magnetite transformation to maghemite. The reaction mixture was then treated with 10 mL of 30% ammonia solution until the pH reached 12, and we continued stirring overnight, resulting in a black-colored precipitation. After the system reached a precipitation state, the precipitation was allowed to cool and settle at the bottom of the flask at room temperature. A permanent magnet was used to separate the black precipitates from the supernatant. The precipitate was rinsed with distilled water and allowed to dry in open air for 24 h.

### 4.4. Preparation of MOLNB Formulations

The mixture of Epikuron 200 (2.5% *w*/*v*) and palmitic acid (0.5% *w*/*v*) was prepared in ethanol. Then, 400 µL of perfluoropentane was added to 300 µL of the mixture of Epikuron 200 and palmitic acid in an ice bath. Then, 4.8 mL distilled water was added dropwise. After this, the complete system was homogenized for 2 min at 20,000 rpm by using a high-shear homogenizer (ultra-turrax TA 18) until the formation of a nanoemulsion. Thereafter, the nanoemulsion was incubated at 37 °C and saturated with oxygen for 10 min. Then, 400 µL of an aqueous solution of dextran sulfate sodium salt (2% *w*/*v*) was added dropwise as a polymeric shell under oxygen purging. Finally, 5.9 mg (1 mg/mL) and 2.95 mg (0.5 mg/mL) of SPION suspensions were added to NB under stirring, to obtain MOLNBs of two different concentrations [25]. MOLNBs containing 1 mg/mL and 0.5 mg/mL SPIONs correspond to around 25.76 % *w*/*w* and 14.78 % *w*/*w* SPION concentrations and were used directly without separating uncoated SPIONs.

### 4.5. In Vitro Oxygen Release Study

In vitro study of oxygen release from MOLNBs was performed at different temperatures (i.e., 25 °C, 37 °C, 41 °C). Dialysis bag technique was used for oxygen release: the bag was filled with 3 mL of donor phase, such as MOLNBs. Then, the bag was placed in 50 mL of saline solution (NaCl 0.9% *w*/*v*); the oxygen concentration of the saline solution was previously reduced up to 2.5 mg/L by purging inert gas (nitrogen, argon). The oxygen release by diffusion was monitored for 24 h using an oximeter (Hach.) HACH HQ 40D is a digital oximeter connected with a LDO 101 digital, luminescent/optical dissolved oxygen (LDO) probe. We set a 30 min time interval for determining oxygen release, and every 30 min, the instrument recorded the oxygen concentration in the sample [28].

### 4.6. Physicochemical Characterization of OLNBs, OLNDs and MOLNBs

The mean diameter, polydispersity index (PDI) and ζ potential were determined by dynamic light scattering spectroscopy (DLS) at room temperature. The samples were diluted with distilled water in an electrophoretic cell. Each determined value was the average of five reciprocals for diameter measurement and 10 reciprocals for ζ potential determination; a 15 V/m electric field was used for ζ potential determination. The viscosity and osmolarity were determined at room temperature by using a capillary viscosimeter and osmometer, respectively. 

### 4.7. Morphological Evaluation

The morphological examination of OLNBs, OLNDs and MOLNBs was carried out by transmission electron microscopy (TEM) in conventional mode, by using a JEOL 2200FS microscope working at 200 kV. MOLNBs were also investigated by high-angle annular dark-field scanning transmission electron microscopy (HAADF-STEM). To minimize the radiation damage from the electron beam, the HRTEM images were acquired using a very low beam current and low exposure time.

### 4.8. Magnetic Measurements and Hyperthermic Properties 

The magnetic properties of OLNBs, SPIONs and MOLNBs were measured at room temperature by an alternating gradient force magnetometer. The magnetization cycles were performed by applying a maximum magnetic field μ_0_H = 1.8 T. 

Calorimetric measurements using an AC commercial applicator were used to evaluate the hyperthermia properties (nanoScale Biomagnetics DM100, Zaragoza, Spain). The specific needs involved in the process of applying a magnetic field and in measuring and analyzing the results were thoroughly examined and approached one at a time, leading to an integrated and final solution that ensured the highest standards in magnetic hyperthermia research.

MOLNBs containing two different low concentrations of SPIONs (0.5 and 1 mg/mL) were tested by means of a DM100 nanoScale Biomagnetics apparatus for the measurement of magnetic hyperthermia. They were exposed for 30 min to an RF alternating magnetic field with amplitude = 300 Oe and frequency = 429 kHz. To avoid coupling with the RF field, the temperature increase was measured with an optical fiber thermometer. Based on a report from the manufacturer, an uncertainty level of ±10% in the temperature measurements was assumed.

The specific absorption rate (SAR) in W/g of the formulation was calculated by the following equation:

$$SAR=\frac{c\cdot \rho }{C}\frac{\mathrm{dT}}{\mathrm{dt}}$$

where c is the specific heat capacity of the sample (assumed to be 4.18 J/g °C), ρ the density of the solvent (equal to 1 g/mL), C the concentration of the magnetic NB in the solvent (1 g/L and0.5 g/L) and dT/dt the heating rate of the sample, calculated by fitting its temperature increase ΔT with a linear trend at an early time point.

### 4.9. Evaluation of Antitumor Effect of Doxorubicin and Curcumin-Loaded OLNDs

TUBO cells, from a cloned rat Her2/neu+ cell line established from lobular carcinoma of a BALB-neuT mouse, were incubated for 48 h in the absence/presence of different concentrations of OLNDs, free chitosan or DOX (either free or carried by OLNDs).

Curcumin-loaded OLNBs were prepared at different concentrations (1-15 uM) and delivered to PC-3 and DU-145 cultured cells. Cell viability was checked by 3-(4,5-dimethylthiazol-2-yl)-2,5-diphenyltetrazolium bromide assay (MTT). All measurements were performed with the same batch, incubation time and microplate reading techniques.

### 4.10. Curcumin Release during HT

The in vitro curcumin release from curcumin-loaded MOLNBs was studied before and after HT using a multi-compartment rotating cell. Curcumin-loaded MOLNBs were placed in the donor chambers. The receiving and the donor chambers were separated by a semi-permeable cellulose membrane (cut-off 14 KDa). The receiving phase for all formulations was 2–Hydroxypropyl-beta-cyclodextrin (5% *w*/*v*). Moreover, 1 mL of sample was withdrawn at fixed times during the experiment, which lasted 26 h. The curcumin concentration was evaluated by HPLC (Shimadzu, UV/VIS detector SPD-20AV.)

### 4.11. MRI Testing

MRI experiments were performed in a clinical magnetic resonance (MR) Scanner (3.0 T, Vida, Siemens). MOLNBs were suspended in tubes at different concentrations of SPIONs, such as 2.5, 2, 1, 0.5 mg/mL. The tubes were placed into the MR scanner and several MR sequences were run. We acquired multiple spin echo varying 10 times the echo time (TE) in steps of 15 ms (max 150 ms, the repetition time was 3000 ms, TR, and the total duration of the measurement was around 20 min). We repeated the experiment 3 times, computing means and SD. To measure signal intensity, manually drawn regions of interest were used for each sample in DICOM viewer software. The data were fitted in Microsoft Excel 2010 with an exponential curve to obtain T_2_ estimates.

### 4.12. Magnetic Field and US Imaging Monitoring

Suspension of MOLNBs was injected into a plastic cylinder and sonicated. Two permanent cylindric magnets (diameter = 6 mm, height = 0.75 mm) of neodymium covered with Ni-Cu-Ni (https://calamite.org, accessed on 26 July 2021) were located on the cylinder wall (see Supplementary Figure S3) and the magnetic fields were calculated by using Python package Magpylib [39]. B-mode US imaging was performed to study the reaction of MOLNBs to the external magnetic field. MOLNBs (at concentration 1 × 10^10^ NB/mL) were injected in the plastic cylinder containing demineralized water with a syringe, through a glass tube. The experiment was carried out at a temperature of 25 °C. MOLNBs were sonicated using US clinical equipment (MyLab™25Gold Esaote, Genova, Italy), connected to a linear array transducer (LA523, 7.5 MHz central frequency, Esaote, Genova, Italy) operating in B-mode (small parts imaging preset). B-mode cineloops (30 s) were acquired and recorded for postproduction both in the absence and in the presence of the magnets. Snapshots from cineloops were extracted at different time frames (5, 15, 30 s) after the initial injection in the different configurations.

## 5. Patents

YES: A NANOSTRUCTURE FOR THE VEHICULATION OF GAS AND/OR ACTIVE INGREDIENTS AND/OR CONTRAST AGENTS AND USES THEREOF, PCT EXTENSION, WO2015/028901 A1.

## Figures and Tables

**Table 1:** Different oxygen-loaded nanosystems. OLNB: oxygen-loaded nanobubbles; OLND: oxygen-loaded nanodroplets.

Shell Polymer	Perfluoropentane C5F12 (PFP) Core	Decafluoropentane C5H2F10 (DFP) Core
Chitosan/Dextran/Dextran sulfate	OLNB	OLND

**Table 2:** T_2_ signal values obtained from curve fitting.

Concentration (mg/mL)	Blank	0.5	1	2	2.5
T_2_ (ms)	2000	58.82	55.55	30.30	29.41

## Data Availability

The data presented in this study are available on request from the corresponding author.

## Supplementary Materials

The following are available online, Figure S1: Graphical representation of the mechanism of oxygen release from OLNBs core: by the application of an external stimulus, i.e., US (a) or by passive diffusion (b), Figure S2: (a) Signal intensities of blank OLNBs at different TE value and (b) signal intensity of 0.5, 1, 2.0 and 2.5 mg/mL. SPIONs concentration on MOLNBs at different TE value as a function of echo delay time. T2 is inversely proportional to concentration of SPIONs loaded on MOLNBs, increased concentration shows a sharp reduction in the signal intensity. The error bars show SD. Figure S3: The setup used for the evaluation of the response of MOLNBs: (a) two magnets positioned on the side of the cylinder wall, at 4 cm distance from each other; (b) projections of magnetic field lines in the XY and XZ plane.
