# Supplementary material for: Step-by-Step Design of New Theranostic Nanoformulations: Multifunctional Nanovectors for Radio-Chemo-Hyperthermic Therapy under Physical Targeting
Source: Molecules. 2021 Jul 29;26(15):4591. doi: 10.3390/molecules26154591 (PMC8348950; doi:10.3390/molecules26154591)
Supplement: Supplementary file 1 [file molecules-26-04591-s001.zip › molecules-1293519-supplementary.pdf]

Supplementary materials

"Step-by-Step Design of New Theranostic Nanoformulations: Multifunctional Nanovectors for Radio-Chemo-Hyperthermic Therapy under Physical Targeting"

Mechanism of oxygen release from OLNBs

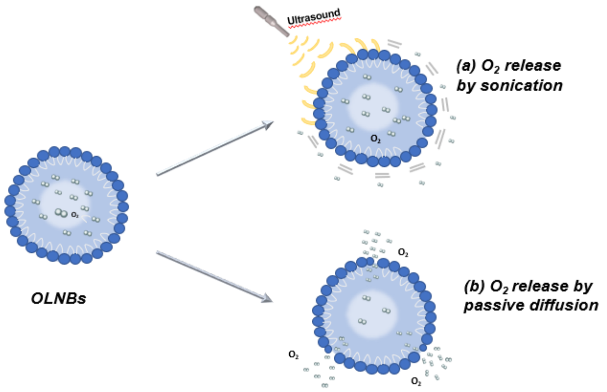

Figure S1. Graphical representation of the mechanism of oxygen release from OLNBs core: by the application of an external stimulus, i.e., US (a) or by passive diffusion (b).

Oxygen Diffusion for Dextran-shelled OLNDs

Effective oxygen diffusion from OLNDs in tissues was confirmed by *in vivo* experiments performed by transcutaneous oximetry ($tcpO_2$) and by photoacoustic imaging system on mouse legs. Details can be found in [30]. In the first case $tcpO_2$ was measured using a transcutaneous oximeter on the shaved abdomens of 8 mice before and after topical treatment with OLNDs in gel formulation followed by sonication (f= 1MHz, P=5 W, t=30 s). Significant increases of $tcpO_2$ (up to 5-fold) were measured in the following 15 minutes and the effects were still evident after 1 hour.
In the second case the oxygen release from OLNDs was compared with that of empty NDs and saline fully saturated with oxygen when topically administered on the shaved hind limbs of 9 anesthetized mice. Oxy-Hb and deoxy_Hb were visualized by Vevo LAZR Photoacoustic Imaging System and the oxygenation effect of OLNDs proved effective and sustained up to 1 hour.

Magnetic Characterization of MOLNBs

Transverse relaxation (T_2) values were obtained from exponential fitting of the signal The signal intensity dependency on echo time (TE) is shown in Supplementary Figure 2.

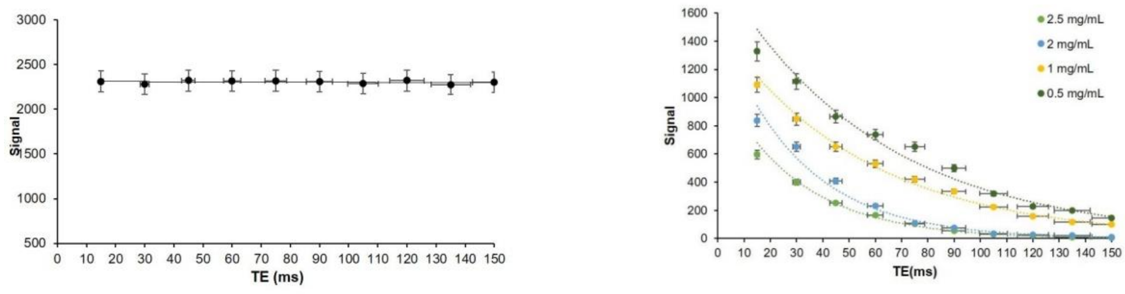

(**a**) (**b**)

Figure S2. (**a**) Signal intensities of blank OLNBs at different TE value and (**b**) signal intensity of 0.5, 1, 2.0 and 2.5 mg/mL. SPIONs concentration on MOLNBs at different TE value as a function of echo delay time. T_2 is inversely proportional to concentration of SPIONs loaded on MOLNBs, increased concentration shows a sharp reduction in the signal intensity. The error bars show SD.

Magnetic Field and US Imaging Monitoring

Supplementary Figure 3 shows a configuration obtained by positioning two permanent magnets on one side of a plastic container (Figure S3a) and the lines of the magnetic field generated (Figure S3b), using [39].

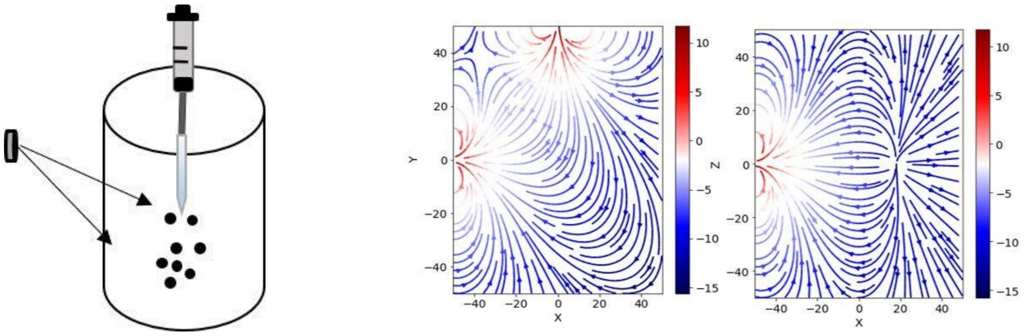

(**a**) (**b**)

Figure S3. The setup used for the evaluation of the response of MOLNBs: (**a**) two magnets positioned on the side of the cylinder wall, at 4 cm distance from each other; (**b**) projections of magnetic field lines in the XY and XZ plane.

30. Prato, M.; Magnetto, C.; Jose, J.; Khadjavi, A.; Cavallo, F.; Quaglino, E.; Panariti, A.; Rivolta, I.; Benintende, E.; Varetto, G.; et al. 2H,3H-Decafluoropentane-Based Nanodroplets: New Perspectives for Oxygen Delivery to Hypoxic Cutaneous Tissues. *PLOS ONE* **2015**, *10*, e0119769, doi:10.1371/journal.pone.0119769.
39. Ortner, M.; Coliado Bandeira, L.G. Magpylib: A Free Python Package for Magnetic Field Computation. *SoftwareX* **2020**, *11*, 100466, doi:10.1016/j.softx.2020.100466.
